# Current News

**Published:** 1895-08

**Authors:** 


### Current News.

### AMERICAN DENTAL ASSOCIATION.

The Thirty-fifth Annual Meeting of the American Dental Association will be held at Asbury Park, New Jersey, commencing Tuesday, August 6, 1895, and continuing for four days.

Railroad rates.—A rate of *a fare and one-third for the round trip*, upon the "certificate plan," has been secured. In order to get this reduction, full fare must be paid in going to the meeting, a *receipt* being obtained therefor from the ticket agent at the *starting-point*. If travelling over more than one line, secure a certificate over each line, or have the ticket agent at the starting-point name in the receipt the different roads over which the ticket is good. This receipt (certificate) must be countersigned by the Secretary of the Association, and entitles the holder to return for one-third fare. Arrangements have been made to have the joint agent of the railroads present at Asbury Park on Wednesday, August 7, and it is desirable that all who intend to attend the meeting shall be in attendance, so that their railroad certificates can be passed upon at that time. No rates have been granted over the lines comprised in the Western Passenger Association. Members residing west of Chicago should secure the best rates they can to the point where they enter the territory in which the reduced rate prevails.

J. N. Crouse,
*Chairman of the Executive Committee.*

2231 Prairie Avenue, Chicago.

---

### HARVARD DENTAL ALUMNI ASSOCIATION.

The twenty-fourth annual banquet of the Harvard Dental Alumni Association was held at the "Thorndike," in Boston, on June 24, 1895, with sixty-three members and guests present. The invited guests were Charles Francis Adams, LL.D.; Hon. Sherman Hoar, United States District Attorney; M. C. Ayres, editor *Boston Advertiser;* Rev. A. E. Winship, editor *Journal of Education;* Victor J. Loring, LL.B., and William M. Conant, M.D., Instructor in Anatomy, Harvard Medical School, all of Boston.

Drs. Charles H. Abbot, '85, and Amos I. Hadley, '91, of Berlin, Germany, Corresponding Secretaries, were present.

The post-prandial exercises were inaugurated by President Dwight M. Clapp in reading a communication from Professor Charles A. Brackett, '73, of Newport, R. I., to the Association, expressing "appreciation of the services of Professor Thomas H. Chandler, Dean of the Dental School, sorrow for his continued illness, and earnest good wishes for his speedy restoration to health." In the form of a resolution it was unanimously adopted, and a copy transmitted to Dr. Chandler.

President Clapp happily introduced the guests, all of those who had accepted invitations being present, and each improved the opportunity to do himself credit by making an excellent speech.

Hon. Sherman Hoar, after much wit, claimed the right for all the professional schools of Harvard University to vote for overseers, saying, "Now, Harvard College controls Harvard University, but Harvard University ought to control Harvard University."

In the absence of Dean Chandler, Professor Thomas Fillebrown described the work and progress of the school during the year past, stating that the entrance examination in '97 would make either Latin or French obligatory, in addition to the present requirements.

The reports of the various officers were made, and the Association elected the following-named officers for the ensuing year:

President, James Shepherd, D.M.D., '85; Vice-President, Frank Perrin, D.M.D., '77; Secretary, Waldo E. Boardman, D.M.D., '86; Treasurer, Washburn E. Page, D.M.D., '77.

*Executive Committee.*—Waldo E. Boardman, D.M.D., '86, chairman; William P. Cooke, D.M.D., '81; Patrick W. Moriarty, D.M.D., '89.

WALDO E. BOARDMAN, D.M.D., '86,
*Secretary.*

BOSTON, July 10, 1895.

---

### WOMAN'S DENTAL ASSOCIATION.

THE Woman's Dental Association met at Dr. Stilwell's office, 1300 Arch Street, May 4, 1895, the President, Dr. Anna Y. Focht, in the chair.

Fannie E. Hoopes, M.D., D.D.S., of Baltimore, was essayist. Subject, "Oral Conditions in Relation to Gastric Diseases." Discussion by all the members present, and by our visitor, Dr. C. N. Peirce.

EMILY W. WYETH,
*Recording Secretary.*

3920 FAIRMOUNT AVENUE, PHILADELPHIA, PA.

---

### ILLINOIS STATE DENTAL SOCIETY.

THE Thirty-first Annual Meeting of the Illinois State Dental Society was held at Galesburg, May 14 to 17, 1895. About two thousand were in attendance. The following-named persons were elected officers for the ensuing year: President, Walter A. Stevens of Chicago; Vice-President, C. R. Taylor, of Streator; Secretary, Louis Ottofy, of Chicago; Treasurer, Edgar D. Swain, of Chicago; Librarian, J. R. Rayburn, of Fairbury; Chairman of Executive Committee, W. A. Johnston, of Peoria. The next meeting will be held at Springfield, May 12 to 15, 1896.

LOUIS OTTOFY,
*Secretary.*

MASONIC TEMPLE, CHICAGO.

---

### ALUMNI ASSOCIATION, UNIVERSITY OF MICHIGAN.

THERE will be a meeting of the Alumni Association of the University of Michigan, Dental Department, at Asbury Park, same time as American Dental Association, August 7, 8, and 9, 1895.

L. L. BARBER,
*Secretary.*

---

### DENTAL SOCIETY OF THE STATE OF NEW YORK.

AT the meeting of the Dental Society of the State of New York held May 8 and 9, 1895, the following officers for the ensuing year were elected: President, Dr. H. J. Burkhart, Batavia; Vice-President, Dr. C. K. Van Vleck, Hudson; Secretary, Dr. C. S. Butler, Buffalo; Treasurer, Dr. John I. Hart, New York; Correspondent, Dr. R. Ottolengui, New York.

